# Blue health among children, adolescents, and youth psychological well-being: a systematic review of swimming and aquatic therapy for mental health

**DOI:** 10.3389/fpsyg.2025.1732568

**Published:** 2026-01-13

**Authors:** Shimeng Huang

**Affiliations:** School of Physical Education, Guangzhou Sport University, Guangzhou, China

**Keywords:** adolescent mental health, anxiety, aquatic therapy, blue health, depression, emotional regulation, psychological wellbeing, psychosocial development

## Abstract

**Introduction:**

The transition from childhood to youth constitutes a pivotal developmental epoch marked by profound physiological, cognitive, and socio-emotional transformations. This period, while dynamic and formative, also ushers in heightened susceptibility to psychological distress, including anxiety, depression, and stress-related disorders.

**Objective:**

Within this context, aquatic therapy and swimming have emerged as promising yet underexplored modalities, offering multifaceted benefits that extend beyond traditional exercise paradigms.

**Method:**

Drawing on interdisciplinary evidence, this review synthesises current empirical and theoretical insights into the efficacy of swimming and aquatic-based interventions for enhancing adolescents’ mental health. By integrating physiological, neurobiological, and psychosocial perspectives, this review elucidates the mechanisms through which aquatic participation mitigates anxiety and depressive symptomatology while strengthening cognitive flexibility, affective balance, and overall well-being.

**Result:**

The synthesis further highlights prevailing gaps in the extant literature, particularly regarding methodological rigor, intervention duration, and the neuropsychological underpinnings of aquatic engagement.

**Conclusion:**

In addressing these lacunae, the review advances a substantial framework for understanding water-based physical activity as both a preventive and rehabilitative instrument within adolescent mental healthcare. Ultimately, this work contributes to the evolving discourse on blue health, positioning swimming and aquatic therapy as potent, evidence-informed avenues for cultivating emotional resilience, self-regulation, and flourishing during adolescence.

## 1 Introduction

Mental health challenges among adolescents have become a critical global concern, with depressive and anxiety disorders now ranking among the leading causes of illness and disability in this age group (WHO, 2024). The World Health Organization estimates that one in seven adolescents aged 10–19 experiences a mental disorder, yet most remain untreated due to stigma, limited access to care, and structural barriers in low- and middle-income settings (WHO, 2024). This alarming prevalence underscores the urgency of identifying accessible, sustainable, and non-stigmatizing interventions that can complement traditional psychological and pharmacological approaches (Patel et al., 2018; Cuijpers et al., 2020).

Children and Adolescents represent a developmental period characterized by heightened emotional sensitivity, identity formation, and social dependency, rendering individuals particularly vulnerable to stress and mood dysregulation (Blakemore and Mills, 2013; Sawyer et al., 2012). While physical activity has consistently been associated with reduced symptoms of depression and anxiety (Biddle et al., 2019; Axelsdottir et al., 2020), much of this evidence is derived from school-based or land-based exercise interventions. The unique potential of aquatic environments integrating physical, psychological, and environmental dimensions, which remains comparatively underexplored, particularly in relation to adolescent populations (White et al., 2020; Gascon et al., 2015). The concept of Blue Health has recently emerged to describe the health-promoting benefits of exposure to or interaction with aquatic environments, such as oceans, lakes, rivers, and swimming pools (Foley and Kistemann, 2015; White et al., 2020). Contact with blue spaces is associated with enhanced psychological restoration, stress recovery, and mood regulation through combined physiological and psychosocial pathways, including parasympathetic activation, endorphin release, and perceived social connectedness (Britton et al., 2021). In addition, water’s physical properties, buoyancy, hydrostatic pressure, and temperature contribute to both physiological relaxation and embodied therapeutic experiences (Silva et al., 2019; van Tulleken et al., 2018). Despite these promising mechanisms, systematic evidence specifically linking aquatic engagement to adolescent mental health outcomes remains limited.

Existing studies suggest that structured swimming, surf therapy, and open-water immersion can alleviate depressive and stress-related symptoms, yet findings are inconsistent, and methodological diversity complicates interpretation (Olive et al., 2022; Burlingham et al., 2022; Carter et al., 2016). While a growing number of programs emphasize water-based therapy as a supportive or preventive measure, a rigorous synthesis focused on adolescents remains lacking. Moreover, most available reviews aggregate evidence across heterogeneous populations or broader “nature-based” interventions, often obscuring age-specific effects and mechanisms (Gascon et al., 2015; White et al., 2020).

To address these gaps, this systematic review examines the psychological outcomes of swimming-based and other aquatic interventions targeting adolescents and youth. Specifically, it explores (1) empirical evidence regarding the impact of such interventions on depression, anxiety, and emotional wellbeing; (2) the underlying physiological, neurobiological, and psychosocial mechanisms proposed to explain these effects; (3) the contextual and methodological factors influencing their effectiveness; and (4) the limitations and future research directions required to strengthen this emerging field. By synthesizing quantitative and qualitative evidence, this review aims to clarify how engagement with blue spaces contributes to adolescent mental health, thereby advancing understanding of nature-based, youth-centered therapeutic approaches within the broader Blue Health paradigm.

### 1.1 Research questions

- What empirical evidence exists on the impact of swimming and aquatic therapy on adolescent mental health outcomes, including anxiety, depression, and emotional regulation?
- Through which physiological perspective do swimming interventions influence children, adolescents, and Youth’s mental wellbeing?
- Which contextual and methodological factors moderate the effectiveness of swimming-based interventions?
- What research gaps and limitations must be addressed to strengthen the evidence base for integrating aquatic programs into adolescent wellbeing and education initiatives?

This review contributes to a growing interdisciplinary conversation on mental health promotion through embodied, nature-based, and educationally aligned interventions by adopting a multidimensional and functional conceptualization of adolescent mental health. In the present systematic review, mental health is not treated as a single clinical construct, but rather as a continuum encompassing emotional, psychological, and social functioning, consistent with the World Health Organization’s definition of mental health as a state in which individuals realize their abilities, cope effectively with everyday stressors, function productively, and contribute to their communities (WHO, 2024). Accordingly, mental health outcomes in this review include both the reduction of negative psychological states, such as symptoms of anxiety, depression, stress, and psychological distress, and the enhancement of positive psychosocial resources, including emotional regulation, self-esteem, self-efficacy, resilience, mood stability, social connectedness, and overall quality of life.

This dual-pathway framing aligns with contemporary models of youth mental health that emphasize wellbeing as more than the absence of psychopathology (Keyes et al., 2002; Keyes and Simoes 2012). Empirical studies in adolescent populations have consistently demonstrated that physical and activity-based interventions exert meaningful effects across these domains, influencing emotional regulation processes (Hofmann et al., 2010), stress responsivity (Gerber et al., 2017), self-concept and self-worth (Babic et al., 2014), and resilience development (Lubans et al., 2016). Within this literature, mental health outcomes are often operationalized using validated psychometric instruments assessing depressive symptoms, anxiety levels, perceived stress, affective states, self-esteem, and psychosocial wellbeing, reflecting both clinical and educational perspectives on adolescent development.

The present review further situates mental health within an ecological and embodied framework, recognizing that interactions between bodily experience, social context, and environmental affordances shape adolescents’ psychological functioning. Aquatic environments, in particular, have been shown to elicit unique psychophysiological responses, including reductions in cortisol, improvements in mood, and enhanced emotional regulation, partly due to sensory immersion, rhythmic movement, and perceived safety and support (Biddle et al., 2019; Niedermeier et al., 2020). Studies on blue-space exposure and water-based physical activity have also linked swimming and aquatic exercise to improved affect, reduced anxiety, and enhanced subjective well-being in young people (Gascon et al., 2015; White et al., 2020).

By systematically examining evidence across experimental and qualitative domains, this review advances theoretical and practical understanding of how aquatic-based interventions contribute to adolescent mental health through both protective and promotive pathways. The findings aim to inform educators, mental health practitioners, and policymakers seeking scalable, inclusive, and non-stigmatizing strategies to support adolescent wellbeing, particularly in school and community contexts where traditional mental health services may be limited or underutilized. Ultimately, this synthesis positions swimming not merely as a form of recreation but as a potential educational and therapeutic practice that bridges physical health, emotional regulation, psychosocial development, and holistic wellbeing during a critical developmental period.

## 2 Literature review

### 2.1 Background and context of young people mental health

Adolescence is a pivotal developmental stage characterised by rapid biological, emotional, and social transformations that profoundly influence long-term mental health trajectories (Shin and You, 2013). The transition from childhood to adulthood introduces heightened academic pressure, identity formation challenges, and evolving peer and family relationships, rendering adolescents particularly susceptible to psychological distress (Mata et al., 2012). Globally, mental health disorders, including anxiety, depression, and attention-deficit/hyperactivity disorder, constitute a substantial public health concern, accounting for approximately 13% of the global disease burden among individuals aged 10–19, with nearly half of all mental disorders emerging by age 14 (Ghazal et al., 2025; Carbonell et al., 2023). Depression alone remains the leading cause of disability among adolescent females and a significant risk factor for early mortality and impaired adult functioning (Axelsdottir et al., 2020; Marciano et al., 2022).

Inadequate engagement in physical activity further compounds these vulnerabilities. Nearly 80% of adolescents worldwide fail to achieve recommended activity levels, a deficit linked to diminished life satisfaction, social withdrawal, and elevated psychological distress (Xu et al., 2025). Conversely, research indicates that structured and socially engaging leisure activities, particularly those involving physical movement, exert a protective effect against affective and anxiety disorders (Timonen et al., 2022; Shin and You, 2013). Within this context, aquatic-based interventions, including swimming, surf therapy, and water-based exercise, have gained attention as multidimensional strategies that combine physiological activation, sensory regulation, and social interaction to foster psychological resilience and emotional balance (Lema-Gómez et al., 2021; Zhou et al., 2025).

The World Health Organization conceptualizes mental health as more than the absence of illness, emphasizing a holistic state in which individuals realize their potential, cope with everyday stress, and contribute productively to their communities (Zhou et al., 2025). This broader psychosocial understanding underscores the importance of integrative, experiential approaches that engage both the body and mind. In educational and developmental contexts, such frameworks position aquatic therapy as an evidence-informed, preventive strategy for promoting well-being, emotional regulation, and cognitive engagement among youth (McKay, 2012; Shin and You, 2013). Despite an expanding body of research linking physical activity to improved mental health, the specific mechanisms through which aquatic environments influence emotional and cognitive outcomes remain underexplored (Zhou et al., 2025). The literature suggests that active participation in swimming and other aquatic programs may elicit distinct psychological benefits compared to passive or sedentary leisure activities by fostering mastery, self-expression, and social belonging (Dupont and Lefevre, 2024). As adolescence represents a sensitive period for the development of lifelong coping strategies, understanding how aquatic interventions can mitigate anxiety, depression, and stress is critical for designing effective, developmentally attuned health and education initiatives (Zhang et al., 2024; Wanka et al., 2025).

### 2.2 Aquatic therapy and swimming interventions

Aquatic therapy encompasses a range of structured and semi-structured water-based activities designed to leverage the therapeutic properties of water buoyancy, hydrostatic pressure, and resistance to facilitate physiological and psychological well-being (Hu et al., 2023; Li et al., 2024).

In this review, aquatic exercise is defined as “an activity that is done in a body of water, such as a pool, a lake, or the ocean” (Hearn et al., 2025; Jackson et al., 2022). The term broadly includes swimming, surf therapy, and other forms of water-based physical activity that involve immersion and movement within aquatic environments. According to the American College of Sports Medicine (ACSM), exercise constitutes “a type of physical activity consisting of planned, structured, and repetitive bodily movement done to improve and/or maintain one or more components of physical fitness” (ASCM, 2018; Riebe et al., 2018). Within this framework, swimming represents “the sport or activity of immersion into water and propelling oneself through water using the limbs.” These operational definitions delineate aquatic exercise as both a physical and environmental experience, integrating intentional movement with the distinctive sensory and physiological qualities of water. The reduced gravitational load in water allows adolescents to engage in physical activity with minimal joint stress, promoting participation and endurance even among those with limited mobility or confidence in traditional exercise contexts (Aryda and Wedastra, 2024). Simultaneously, the sensory and proprioceptive stimulation provided by water immersion can reduce anxiety, induce relaxation, and improve mood regulation (Fairbrass et al., 2020; Zhang et al., 2024).

Evidence suggests that aquatic-based aerobic and mind–body exercises yield substantial reductions in anxiety and depressive symptoms among youth, comparable to or exceeding those of land-based interventions (Barahona-Fuentes et al., 2021; Zhang et al., 2024). The calming and mindful qualities of water immersion are further linked to improved attention, emotion regulation, and resilience against life stressors (Burger et al., 2024). These benefits are consistent with broader evidence supporting the restorative potential of natural “blue spaces,” which have been associated with higher levels of life satisfaction, physical self-perception, and social engagement (Gómez-Paniagua et al., 2025; Cabana et al., 2024). Beyond physical mechanisms, the social dimension of aquatic programs fosters a sense of belonging, teamwork, and peer support, key psychosocial factors that reinforce self-esteem and emotional stability (Shin and You, 2013). For adolescents with neurodevelopmental conditions such as autism spectrum disorder, aquatic therapy provides a low-arousal, sensory-regulated environment that enhances social interaction and motor development (Battaglia et al., 2019; Kalra et al., 2025). The tactile and proprioceptive feedback of water supports adaptive emotional responses and encourages consistent participation, which is essential for long-term mental health benefits (Ogonowska-Słodownik et al., 2024; Koumenidou et al., 2023).

### 2.3 Mechanisms linking aquatic interventions to adolescent mental health

Aquatic therapy operates through interconnected physiological, neurobiological, and psychosocial pathways. Physiologically, buoyancy and water resistance facilitate aerobic conditioning, enhance endorphin release, and improve cardiovascular efficiency, all of which contribute to reduced depressive and anxiety symptoms (Barahona-Fuentes et al., 2021). Neurobiologically, exercise-induced modulation of neurotransmitters (e.g., serotonin, dopamine) and neurotrophic factors (e.g., BDNF) may underpin improved cognitive flexibility and emotional regulation (Belcher et al., 2020). Psychosocially, structured aquatic programs create environments conducive to social interaction, self-efficacy, and intrinsic motivation, which are known mediators of mental well-being (Zhou et al., 2025). Emerging evidence from neuroimaging and behavioral studies suggests that aquatic exercise may enhance neural connectivity in brain regions associated with emotional control and executive functioning (Xu et al., 2025). Such mechanisms highlight the potential for aquatic interventions to serve as both preventive and rehabilitative modalities, particularly within educational and community-based programs. Incorporating neuroscientific methods, such as electroencephalography and longitudinal cohort designs, could further elucidate the causal pathways linking aquatic engagement to improved psychological outcomes (Fairbrass et al., 2020; Maharja et al., 2022).

## 3 The present review

The past decade has witnessed a profound shift in the daily behaviors of adolescents and youth, primarily driven by the rapid expansion of digital technologies. Young people, including children, adolescents, and youth, aged 3–25 according to WHO (2024), now represent the largest consumers of smartphones, social media, video-based platforms, and indoor gaming technologies. While digital engagement offers important educational and social benefits, global evidence consistently shows that excessive screen exposure is contributing to widespread physical inactivity, sedentary lifestyles, and reduced participation in outdoor or nature-based activities among adolescents and young adults. This behavioral shift has coincided with alarming increases in mental health problems, including depression, anxiety, stress, emotional dysregulation, self-harm ideation, and suicide attempts, trends documented across high-, middle-, and low-income contexts.

In response to the global rise in adolescent and youth mental health concerns, researchers have increasingly examined the therapeutic effects of physical activity interventions such as running, football, yoga, and structured exercise programs. More recently, a growing body of literature has highlighted aquatic and water-based activities, including pool swimming, open-water swimming, surf therapy, hydrotherapy, and cold-water immersion, as promising modalities for improving psychological well-being. These interventions integrate physical exercise with sensory stimulation, exposure to the natural environment, and social connectedness factors known to support emotional regulation, resilience, stress reduction, and overall mental health. The emerging field of “blue health” has therefore gained substantial scholarly attention as an innovative and potentially impactful approach to youth mental wellbeing. Despite this progress, existing systematic reviews have several limitations that restrict their applicability to children, adolescents, and youth. Many prior reviews have focused on older adults, clinical populations, individuals with physical or neurodevelopmental disorders, or broad age ranges that obscure youth-specific outcomes. Few synthesize evidence exclusively on adolescents and young adults in the general population, even though this developmental period is uniquely sensitive to mental health risks and lifestyle changes. Furthermore, the literature on water-based interventions has expanded considerably over the past decade, necessitating an updated and youth-focused synthesis of the evidence.

Guided by these gaps, the present systematic review aims to synthesize research published between 2010 and 2025 examining the effects of swimming and other structured water-based interventions on mental health outcomes among adolescents and youth aged 3–25. By integrating evidence across experimental, quasi-experimental, quantitative, and correlational studies, this review provides an understanding of how aquatic activities support mental well-being in this population. The goal is to generate a significant, up-to-date evidence base capable of informing practitioners, educators, policymakers, and researchers seeking effective strategies to address the escalating mental health challenges faced by today’s adolescents and young adults.

## 4 Methods

This systematic review followed the PRISMA 2020 (Preferred Reporting Items for Systematic Reviews and Meta-Analyses) guidelines: the review protocol specified inclusion criteria, search strategy, screening, data extraction, and synthesis procedures.

### 4.1 Eligibility criteria

This systematic review applied clearly defined inclusion and exclusion criteria based on the PICOS framework to ensure that the evidence gathered meaningfully addressed the study’s aim of evaluating the mental health impacts of swimming and aquatic therapy among adolescents and youth (see Table 1). The criteria were developed to reflect current global concerns regarding physical inactivity, the rise of indoor lifestyles, and increasing rates of mental illness, including depression, anxiety, and emotional distress, among adolescents and young adults. Given the growing recognition of “blue health” and aquatic-based activities as potential therapeutic interventions, these criteria ensured the review captured a rigorous and relevant body of evidence.

**Table 1:** PICOS framework with inclusion/exclusion criteria and justifications.

PICOS element	Inclusion criteria	Exclusion criteria
Population	• Adolescents and youth aged 3–25 years. • Mixed-age samples only if data for ages 3–25 can be extracted separately.	• Participants younger than 3 or older than 25. • Mixed-age samples without disaggregated youth data.
Intervention	• Swimming or aquatic-based therapy, including: —pool-based swimming—Open-water/sea swimming—surf therapy—Hydrotherapy or structured aquatic exercise programs • Interventions designed to promote wellbeing, mental health, or psychosocial outcomes.	• Land-based exercise interventions. • Generic physical activity programs not involving water. • Studies assessing only physiological or biomechanical outcomes without mental health measures.
Comparison	• Any comparison group, including: —land-based exercise—no treatment/usual care—wait-list controls—other aquatic interventions • Comparison not required for single-group pre–post designs.	• Comparisons unrelated to mental health or psychosocial outcomes.
Outcomes	• Mental health indicators such as: —depression, anxiety, stress—emotional regulation—mood—self-esteem, resilience—wellbeing, psychological distress • Outcomes measured using validated psychological tools.	• Studies reporting only physical fitness, motor skills, biomechanics, or physiological outcomes. • Behavioral outcomes not linked to mental health (e.g., attendance, discipline).
Study design	• Randomized controlled trials (RCTs) • Quasi-experimental studies • Cross-sectional or correlational studies • Pre–post intervention designs • Mixed-methods and qualitative studies examining psychological outcomes	• Systematic reviews, meta-analyses, commentaries, editorials. • Case studies without empirical data. • Protocols without results.
Setting and duration	• Community, school, clinic, sport, and open-water settings. • Intervention duration of ≥2 weeks.	• Interventions lasting less than 2 weeks or single-session exposure.
Publication type and language	• Peer-reviewed articles • English-language publications	• Non–peer–reviewed sources • Non-English publications

#### 4.1.1 Population

Studies were included if they involved participants aged 3 to 25 years, a range selected to represent adolescents and youth undergoing profound developmental, psychological, and social transitions. This age group is also widely recognized by the World Health Organization as a population at heightened risk of mental health challenges. With global reports indicating an increase in inactivity and escalating mental health concerns among young people, prioritizing this population was essential. Studies involving broader age ranges were included; the range for the 3–25 subgroup was reported separately. Studies focusing on children younger than 3 or adults older than 25, or those that did not allow extraction of youth-specific findings, were excluded to maintain demographic relevance and conceptual coherence.

#### 4.1.2 Intervention

Eligible studies examined swimming or aquatic-based interventions, including pool swimming, surf therapy, hydrotherapy, and open-water or sea swimming. These activities were included because they combine physical activity with exposure to natural or aquatic environments, both of which are known to offer unique psychological benefits. Given the review’s aim to understand how water-based interventions may counter increasing sedentary and indoor behaviors, only studies explicitly involving aquatic activities designed to influence wellbeing or mental health were included. Interventions that did not use water-based activity or that focused exclusively on physiological or motor outcomes without assessing mental health were excluded.

#### 4.1.3 Comparison

Studies employing comparison groups such as land-based exercise, usual care, wait-list controls, or alternative aquatic activities were eligible. Single-arm pre–post studies without comparison groups were also included when they assessed relevant outcomes. Comparisons unrelated to mental health were excluded, ensuring outcomes remained aligned with the review’s central focus on psychological wellbeing.

#### 4.1.4 Outcomes

Included studies were required to assess mental health indicators such as depression, anxiety, stress, mood, resilience, self-esteem, or emotional regulation using validated psychological tools. This requirement ensured that the review synthesized high-quality evidence directly linked to psychological functioning. Studies reporting solely physical fitness, motor coordination, or physiological outcomes were excluded because they do not address the mental health dimensions central to the review.

#### 4.1.5 Study design

To build a comprehensive and diverse evidence base, the review included randomized controlled trials, quasi-experimental studies, cross-sectional studies, pre–post designs, mixed-methods studies, and qualitative research. This range allowed the review to capture both controlled evaluations and naturally occurring experiences of swimming among youth. Reviews, meta-analyses, editorials, protocols, and non-empirical papers were excluded because they do not provide primary data required for systematic analysis.

#### 4.1.6 Setting and duration

Studies conducted in community, school, clinical, recreational, and open-water settings were considered, reflecting the varied environments in which adolescents and youth engage in swimming. Only interventions lasting at least 2 weeks were included to ensure adequate exposure time for psychological change. Short-term or single-session exposure studies were excluded because they lack sufficient duration to assess meaningful mental health effects.

#### 4.1.7 Publication characteristics

Only peer-reviewed articles published in English were included to ensure methodological rigor and analytical feasibility. Non-peer-reviewed sources and articles in other languages were excluded due to challenges in verifying methodological quality and ensuring consistency in analysis.

### 4.2 Information source and search strategy

An extensive and well-documented literature search was conducted across five major databases: PubMed, Web of Science, Scopus, and ProQuest. These databases were chosen for their broad coverage of psychology, public health, health sciences, and behavioral research. The initial search took place from June to September 2025. It focused on peer-reviewed studies published between 2010 and 2025 that examined the link between swimming or other aquatic activities and mental health outcomes among adolescents and youth. The search strategy was developed in stages. First, an exploratory exercise identified key concepts, terms, and keywords from important studies and previous reviews. These helped shape the initial Boolean search strings, which were created beforehand and structured around three main areas: aquatic activities (like swimming, aquatic exercise, and hydrotherapy), population (such as adolescent, youth, and young people), and mental health outcomes (including mental health, well-being, depression, anxiety, and stress). Boolean operators (AND/OR) were used to combine these areas into effective search strings. Minor changes were made to align with the syntax and vocabulary of each database, such as using Medical Subject Headings (MeSH) in PubMed. All retrieved records were entered into reference management software, where duplicates were removed before screening titles and abstracts.

After peer review, concerns emerged that the original search strategy might have missed studies framed as nature-based or blue-space interventions. This included studies describing wild swimming, open-water swimming, sea swimming, or cold-water immersion that were not explicitly labeled as “aquatic exercise” or “swimming interventions.” In response, the search strategy was updated by broadening the aquatic exposure area of the Boolean search string. Additional keywords and phrases relating to environmental context and therapeutic framing were added, such as blue space, nature-based intervention, outdoor swimming, wild swimming, open-water swimming, sea swimming, and cold-water immersion (see Table 2). These new terms were included using OR operators and combined with the existing population and mental health areas using AND operators, ensuring consistency with the original search while improving sensitivity.

**Table 2:** Literature search strategy.

Concept domain	Extensive search keywords (combined with OR within domain)	Boolean operator between domains
Swimming and aquatic-based interventions	swim* OR swimming OR swimmer* OR “aquatic activity” OR “aquatic activities” OR “aquatic exercise” OR “aquatic exercises” OR “aquatic intervention” OR “aquatic interventions” OR “aquatic therapy” OR “aquatic therapies” OR hydrotherapy OR “water-based activity” OR “water-based activities” OR “water-based exercise” OR “water-based exercises” OR “water-based intervention” OR “water-based interventions” OR “aqua therapy” OR “aqua therapies” OR “aqua exercise” OR “aqua exercises” OR “pool-based activity” OR “pool-based activities” OR “pool-based exercise” OR “pool-based intervention” OR “water sport” OR “water sports” OR “structured swimming program” OR “recreational swimming” OR “therapeutic swimming”	AND
Mental health and psychological outcomes	“mental health” OR “mental wellbeing” OR “mental well-being” OR “mental wellness” OR “psychological health” OR “psychological wellbeing” OR “psychological well-being” OR “emotional health” OR “emotional wellbeing” OR “emotional well-being” OR anxiety OR anxious OR depression OR depressive OR stress OR distress OR “psychological distress” OR mood OR affect OR “affective state” OR “emotional regulation” OR “emotion regulation” OR “self-esteem” OR “self concept” OR “self-concept” OR “self efficacy” OR “self-efficacy” OR resilience OR coping OR “coping skills” OR “mental resilience” OR “life satisfaction” OR “subjective wellbeing” OR “quality of life” OR “psychosocial wellbeing” OR “psychosocial health” OR loneliness OR “social connectedness” OR “social wellbeing”	AND
Population: adolescents, youth, and young people	adolescent* OR adolescen* OR adolescence OR teen* OR teenager* OR youth OR “young people” OR “young person” OR “young adults” OR “early adulthood” OR “emerging adults” OR “school-aged children” OR “secondary school students” OR “high school students” OR “middle school students” OR “college students” OR “university students” OR “postsecondary students” OR “late adolescents”	AND
Intervention, program, and exposure terms	intervention* OR program* OR programme* OR training OR “exercise program” OR “physical activity program” OR “sports-based intervention” OR “health promotion program” OR “mental health intervention” OR treatment OR therapy OR exposure OR participation OR engagement OR implementation OR evaluation	AND
Study design and methodological filters	trial OR “clinical trial” OR “controlled trial” OR “randomized controlled trial” OR RCT OR randomi* OR experiment* OR “quasi-experimental” OR “pretest-posttest” OR “pre-post study” OR longitudinal OR “follow-up study” OR cohort OR “mixed methods” OR qualitative OR quantitative	AND
Context and setting (optional but included for sensitivity)	School* OR community OR clinical OR healthcare OR recreational OR sport* OR “physical education” OR “after-school program” OR “community-based program” OR “youth program”	Optional AND

To address the reviewer’s concerns and identify newly published studies, an updated search was conducted in December 2025 using revised Boolean search strings across all five databases. This update served two purposes: first, to include recent publications that emerged after the first search window, and second, to identify additional eligible studies that may have been overlooked due to earlier terminology constraints. The records retrieved in December 2025 were screened using the same predefined inclusion and exclusion criteria. They underwent the same title, abstract, and full-text evaluation procedures as studies found in the initial search. This iterative and open approach ensured that the final evidence base demonstrated both rigorous methods and responsiveness to peer-review feedback, while maintaining consistency in how studies were identified, retrieved, and evaluated throughout the review process.

A combination of controlled vocabulary terms and free-text keywords was employed to ensure the inclusion of both broad and specific studies. To address the heterogeneity of the included studies and ensure that relevant literature framed within the broader “blue health” context was captured, the original search strategy was expanded to include a broader range of program-based, therapeutic, and environment-specific terms. Beyond the general terms such as “swimming,” “aquatic sports,” and “aquatic exercises,” additional keywords were incorporated to reflect structured interventions, clinical programs, and blue-space exposures relevant to the mental well-being of individuals aged 3–25. These included terminology commonly used in rehabilitation, physiotherapy, mental health promotion, and environmental health research, such as “aquatic therapy,” “hydrotherapy,” “water-based therapy,” “aquatic physiotherapy,” “aquatic rehabilitation,” “therapeutic swimming,” “water-based intervention,” “pool-based program,” and “blue space exposure.” Mental health–related terms were also broadened to include concepts such as psychological well-being, emotional well-being, resilience, stress reduction, and affect, ensuring that studies addressing a wider range of youth mental health outcomes were identified. Age-related descriptors were added to ensure that studies focusing on adolescents and youth aged 3–25 were adequately represented. This search strategy yielded 490 potentially relevant records, which were subsequently screened against the predefined eligibility criteria.

### 4.3 Screening process

**Figure 1:**
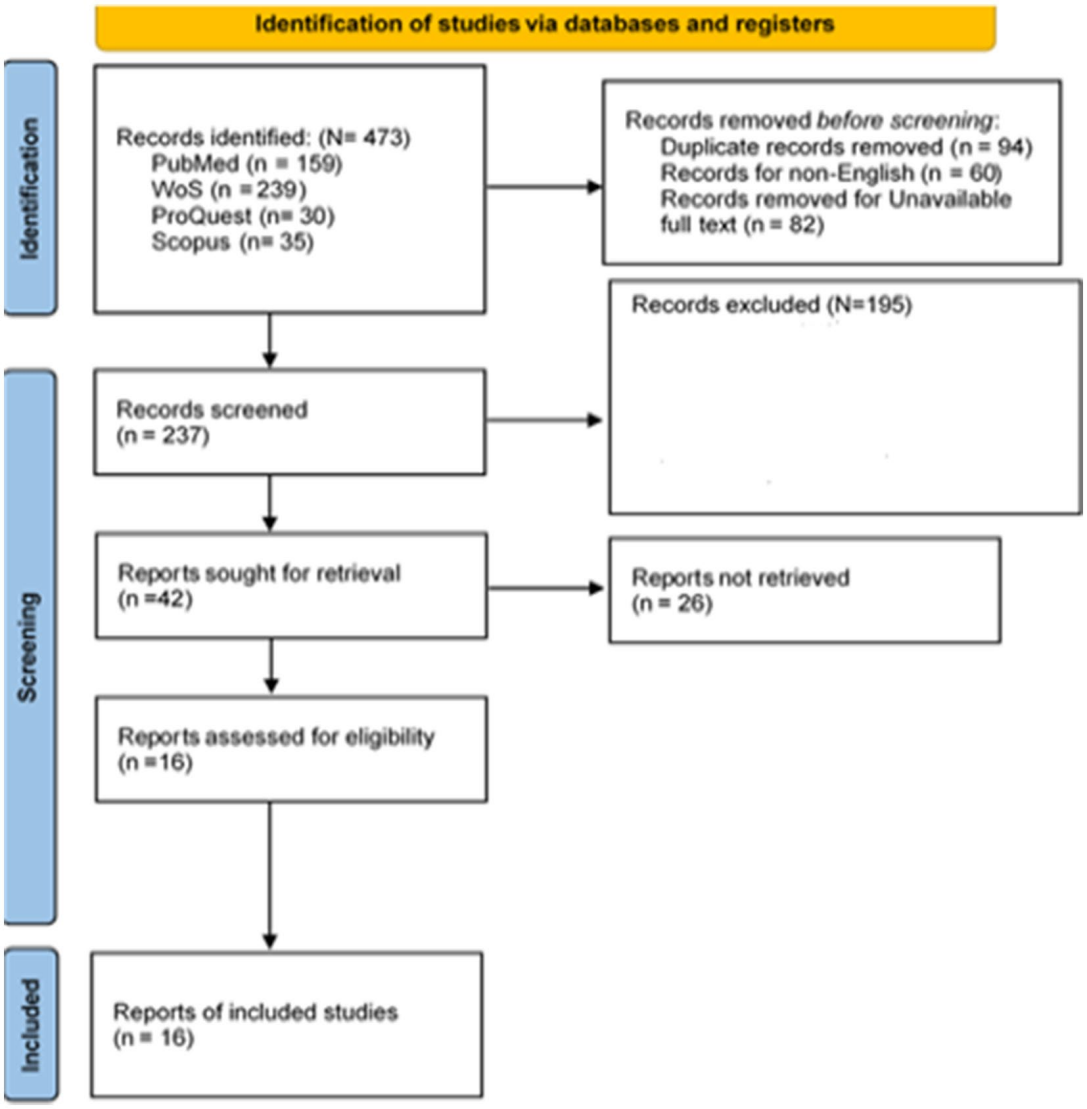
PRISMA flow diagram (Page et al., 2021).

All retrieved titles and abstracts were systematically screened to assess their relevance to swimming-based interventions targeting mental health outcomes among adolescents. The full-text articles of potentially eligible studies were then reviewed in detail according to the predefined eligibility criteria outlined above. After removing duplicate records and excluding studies that did not meet the inclusion criteria, eight studies were deemed eligible and included in the final qualitative synthesis (see Figure 1).

### 4.4 Data extraction

Data extraction was conducted using a structured and transparent process to ensure comprehensive and accurate capture of all information relevant to the predefined inclusion and exclusion criteria. Given that this systematic review focused on adolescents and youth aged 3–25 years engaged in swimming or aquatic-based interventions targeting mental health outcomes, the extraction framework was explicitly developed in accordance with the PICOS criteria and informed by established theoretical perspectives in blue-health and physical activity research. Although the single author performed the initial data extraction, two external, independent scholars were formally engaged to provide methodological oversight and verify all extracted data. Both scholars are experienced researchers with demonstrated expertise in conducting and publishing systematic reviews and meta-analytic studies in public health, mental health, and physical activity interventions.

The external scholars independently reviewed the extracted study characteristics, intervention descriptions, outcome measures, and methodological details to assess accuracy, completeness, and consistency with the source materials. Their role was not limited to procedural checking but extended to critical appraisal of interpretive decisions, particularly in cases where study reporting was ambiguous or where classification of outcomes required theoretical judgment. Discrepancies or uncertainties were discussed collaboratively until consensus was reached, thereby minimizing subjective bias and enhancing the credibility and reproducibility of the review process. The involvement of these independent experts ensured that data extraction and subsequent quality assessment were grounded in disciplinary expertise and aligned with current standards for high-quality systematic reviews, despite the review being authored by a single researcher.

For each eligible study, comprehensive information was extracted regarding research design, methodological characteristics, and intervention setting to document the diversity of study types permitted under the inclusion criteria—such as randomized controlled trials, quasi-experimental designs, cross-sectional studies, pre–post studies, mixed-methods, and qualitative inquiries. Particular attention was directed toward sample characteristics, including sample size, age range, mean age, gender composition, and baseline mental health profile. These data were essential for confirming that studies met the demographic focus on adolescents and youth or, where age ranges were broader, for extracting meaningful youth-specific data.

Given that swimming and aquatic-based interventions form the core of the review, detailed information was recorded on the structure, type, and delivery of each intervention. This included the form of aquatic activity (pool swimming, surf therapy, hydrotherapy, or open-water swimming), frequency and duration of participation, total intervention period, and any programmatic features designed to promote mental health or well-being. In addition to documenting intervention design, contextual and environmental characteristics were extracted to capture the unique ecological dimensions of blue-space interventions. This included the type of aquatic environment, water conditions, natural versus artificial settings, accessibility, safety management, and environmental quality. Because blue-space interventions inherently occur within vulnerable aquatic ecosystems, data were also extracted, where available, on the environmental pressures associated with each intervention. This encompassed potential ecological disturbances such as overcrowding, prolonged water use, disruption to natural water bodies, and any environmental management strategies reported by the authors. Extracting these variables addressed an important gap in previous work and responded directly to emerging concerns regarding the sustainability and ecological impacts of blue-health practices.

Mental health outcomes remained a central component of the extraction process. Data were gathered on depression, anxiety, stress, mood, emotional regulation, self-esteem, resilience, and broader indicators of psychological well-being, provided these were assessed using validated psychological tools. Where reported, quantitative metrics such as effect sizes, confidence intervals, *p*-values, and follow-up outcomes were extracted to support a meaningful synthesis of intervention effects. In addition, feasibility indicators such as participant adherence, program acceptability, engagement levels, drop-out rates, recruitment challenges, and perceived benefits or barriers were documented. These elements are essential to understanding the practicality of implementing aquatic interventions for adolescents and youth within real-world settings, especially given rising rates of physical inactivity and mental health challenges associated with digitalized and indoor lifestyles.

Following extraction, all data were independently reviewed by the external scholars to ensure accuracy and completeness. Any inconsistencies were resolved through discussion and consensus. The final dataset was synthesized narratively to identify convergence and divergence across studies, with specific attention to methodological quality, environmental context, and the extent to which different aquatic environments supported or constrained mental health outcomes. This approach enabled a holistic interpretation of how swimming and blue-space interventions influence adolescent and youth mental health across diverse ecological and programmatic settings, while rigorously adhering to the inclusion and exclusion criteria guiding the review.

### 4.5 Risk of bias assessment

The risk of bias for each study was evaluated independently by two reviewers using criteria adapted from the CONSORT reporting guidelines (Mehr, 2011) (see Table 3). Each study received a score on a 6-point scale, with each criterion receiving a 1 if clearly reported and implemented, or a 0 if missing or insufficiently described. The assessment considered the following aspects: (a) whether groups were equivalent at baseline based on key demographic or clinical characteristics, determined through reported stratified baseline data; (b) whether a control group was included and the method of randomization was clearly outlined and appropriately executed (e.g., sealed envelopes or computerized methods); (c) whether the study reported a sample size or power calculation and demonstrated sufficient statistical power to detect intervention effects; (d) whether outcome assessors were blinded to group allocation at both baseline and follow-up; (e) whether participant retention was reported and whether at least 80% of the sample completed follow-up assessments; and (f) whether statistical analyses accounted for any initial group differences. Based on total scores, studies were categorized as high risk of bias (0–2), moderate risk (3–4), or low risk (5–6).

**Table 3:** Risk of bias assessment.

Studies	(i) Were the groups comparable at baseline on key characteristics?	(ii) Did the study include a control group, and was the process of randomization clearly described and adequately carried out?	(iii) Did the study report a power calculation, and was the study adequately powered to detect intervention effects?	(iv) Were the assessors blinded to treatment allocation at baseline and posttest?	(v) Did at least 80% of participants complete follow-up assessments?	(vi) Did the study account for potential differences at baseline in the analyses	Total
Abdelrahman et al. (2024)	1	1	0	1	1	1	5
Hattabi et al. (2022)	1	1	0	0	1	1	4
Mills et al. (2020)	1	0	1	1	1	1	5
Yu et al. (2024)	1	1	1	1	1	1	6
Köroglu and Yigiter (2016)	1	1	1	0	1	1	5
Massey et al. (2025)	1	0	1	1	1	1	5
Harper et al. (2025)	1	1	1	1	1	1	6
Silva et al. (2019)	1	1	1	1	1	1	6
Forsten and Wetherell (2025)	1	1	0	1	1	1	5
Zulkifli and Bachtiar (2025)	1	1	1	0	1	1	5
Mills et al. (2020)	1	1	1	1	1	1	6
Yu et al. (2024)	1	0	1	1	1	1	5

Overall, the studies included in this review demonstrated a generally low risk of methodological bias, as outlined in Table 2 above in the online Supplementary materials. The two independent reviewers demonstrated high consistency (94% agreement) in their assessments, and any minor discrepancies were resolved through discussion. Most studies provided sufficiently detailed baseline characteristics, allowing clear comparisons between the intervention and control groups. The majority also incorporated an appropriate control or comparison condition, and several employed randomized controlled designs with adequately described procedures for random assignment. Several quasi-experimental studies were also included, but these typically employed structured comparison groups and clearly defined intervention protocols, thereby strengthening their methodological rigor. Although only a few studies reported formal power calculations, sample sizes were generally adequate in relation to their stated research aims. Several studies described procedures to ensure that assessors were blind to group allocation, thereby reducing the likelihood of measurement bias. Adherence and retention rates were also reported in most cases, with follow-up completion rates meeting acceptable thresholds for intervention research. In addition, the majority of studies accounted for baseline differences in their analyses, either through statistical adjustment or by demonstrating group equivalence at the outset. Collectively, these features suggest that the evidence base included in this review is methodologically sound and offers credible insights into the effects of aquatic-based interventions on adolescent and youth mental health.

Furthermore, all four qualitative studies chosen for this systematic review were carefully assessed by two independent reviewers using the Joanna Briggs Institute (JBI) Critical Appraisal Checklist for Qualitative Research. This ensures quality and credibility before they are included. The checklist has ten criteria that evaluate the clarity, openness, and reliability of qualitative studies. It examines how well the research objectives, methods, and underlying beliefs align; the suitability of the data collection and analysis methods; how well participants’ voices are interpreted and represented; the impact of the researchers; and compliance with ethical standards. Each criterion will be rated as Yes, No, or Unclear. Any disagreements between reviewers will be settled through discussion or by bringing in a third reviewer for agreement. Studies that receive a moderate to high-quality rating (scores of 7–10) will be included in the synthesis. Lower-quality studies will be noted but excluded from the primary analysis. This careful evaluation process makes sure that only reliable evidence is used in the synthesis and interpretation of findings in this systematic review (see Table 4).

**Table 4:** JBI quality appraisal report of qualitative study (*n* = 4).

Items	Yes	No	Unclear	Not applicable
1. Is there congruity between the stated philosophical perspective and the research methodology?	□	□	□	□
2. Is there congruity between the research methodology and the research question or objectives?	□	□	□	□
3. Is there congruity between the research methodology and the methods used to collect data?	□	□	□	□
4. Is there congruity between the research methodology and the representation and analysis of data?	□	□	□	□
5. Is there congruity between the research methodology and the interpretation of results?	□	□	□	□
6. Is there a statement locating the researcher culturally or theoretically?	□	□	□	□
7. Is the influence of the researcher on the research, and vice versa, addressed?	□	□	□	□
8. Are participants, and their voices, adequately represented?	□	□	□	□
9. Is the research ethical according to current criteria, or, for recent studies, is there evidence of ethical approval by an appropriate body?	□	□	□	□
10. Do the conclusions drawn in the research report flow from the analysis or interpretation of the data?	□	□	□	□

Based on Table 4, across the four included cross-sectional studies, three demonstrated high methodological qualities, meeting nearly all JBI criteria. These studies clearly defined inclusion criteria, thoroughly described the participants and study context, and used validated, reliable tools to measure exposures and outcomes. Confounding factors were explicitly identified, and appropriate strategies such as statistical adjustments or subgroup analyses were applied. The use of robust statistical techniques further strengthened the validity of their findings (Zulkifli and Bachtiar, 2025; Mullooly and Colbert, 2024). In contrast, one study exhibited moderate quality due to limitations in exposure measurement and confounding control (Burlingham et al., 2022). Although the sample and setting were adequately described and outcome measures were reliable, the study did not clearly report how exposure variables were validated, nor did it outline strategies to mitigate confounding factors. These gaps reduce confidence in the internal validity of the results. Overall, the cross-sectional evidence base is strengthened by the predominance of high-quality studies, though isolated methodological gaps remain.

### 4.6 Quality of the evidence

Two independent researchers conducted all quality assessments to ensure inter-coder reliability. Any inconsistencies in their initial judgments, including those related to risk of bias and the quality of evidence, were resolved first through consensus discussion. In cases of persistent disagreement or ambiguity in the application of criteria, a third external reviewer was consulted to reach a final decision (Guyatt et al., 2011a,b,c).

The overall confidence in the evidence synthesized from the 12 experimental studies was formally evaluated using the Grading of Recommendations Assessment, Development and Evaluation (GRADE) framework (Guyatt et al., 2008; Balshem et al., 2011). For each primary outcome, the quality of evidence was graded as high, moderate, low, or very low. The standard GRADE methodology was applied, which involved assessing four key factors: risk of bias, inconsistency, indirectness, and imprecision (Guyatt et al., 2011a,b,c). The evidence from randomized controlled trials began with a default high-quality rating. This rating was then subject to potential downgrading based on pre-specified criteria. The indirectness domain was not considered for downgrading, as all included studies were directly relevant to the review’s specified population, intervention, comparator, and outcome questions (Guyatt et al., 2011a,b,c). Evidence was downgraded by one level for risk of bias if more than 50% of participants contributing to an outcome were from studies with a PEDro score of 10 or fewer out of 12, indicating limitations in study design or execution (Guyatt et al., 2011a,b,c). A downgrade for imprecision was applied if the total number of participants across all studies in a given meta-analysis was less than 400 (Guyatt et al., 2011a,b,c). See Supplementary Tables A, B.

## 5 Results

### 5.1 Study characteristics

Following a rigorous screening and synthesis process, a total of sixteen studies were included in this systematic review. Collectively, these studies reflect a growing international research interest in swimming and aquatic-based interventions as mechanisms for promoting mental health and psychological well-being across different developmental stages. Of the sixteen studies, twelve employed experimental or intervention-based designs. At the same time, four adopted quantitative or qualitative approaches to generate an in-depth understanding of participants’ lived experiences and perceived mental health benefits associated with swimming (see Table 5).

**Table 5:** Study characteristics of included studies (*n* = 16).

Study	Country	Aim of study	Setting	Sample size (N)	Age group	Design/method
Huque and Yue (2025)	UK	Explore wild swimming groups as “ecologies of care” and their well-being impact	Wild/open-water swimming groups	NR	Youth	QUALI
McDougall et al. (2022)	UK	Examine health, well-being, place, and risk in freshwater wild swimming	Freshwater lakes/rivers (wild swimming sites)	12	Youth (18)	QUALI
Abdelrahman et al. (2024)	Egypt	Compare aquatic vs. aerobic exercise for dysmenorrhea and QoL	Clinical/physiotherapy center	60 (Group A = 30, Group B = 30)	Adolescent females (M = 17.37)	RCT
Zulkifli and Bachtiar (2025)	Malaysia	Assess swimming’s impact on cognitive and mental health in PE trainees	University setting	30	Young adults (undergraduate Students)	PRE-POST
Forsten and Wetherell (2025)	UK	Explore cold-water sea swimming and daily mental health indicators	Open sea swimming	*N* = 13	female youths (age of 18 years)	QEXP
Taylor et al. (2025)	UK/Ireland	Explore sea-swimming as an intervention for youth with mental health challenges	Sea-swimming youth program	*N* = 14	Young people	QUALI
Hattabi et al. (2022)	Tunisia	Assess swimming-based treatment for ADHD in children	Sports/clinical intervention center	*N* = 40, (5 females and 35 males) diagnosed with ADHD	Children (6–12 yrs)	RCT
Mills et al. (2020)	Australia	Evaluate hydrotherapy for ASD-related behaviors and wellbeing	Hydrotherapy pool	(*n* = 8)	6–12 years and diagnosed with ASD	RCT
Yu et al. (2024)	China	Investigate parent-accompanied swimming on physical and cognitive development	Kindergarten/community pool	N 36 boys the traditional physical exercise group (TP, *n* = 12), the accompanied swimming group (AS, *n* = 12) and the independent swimming group (IS, *n* = 12)	mean age 3.56 ±	PRE-POST
Köroglu and Yigiter (2016)	Turkey	Test the effects of swimming training on stress levels	School/sports facility	*N* = 60	students ages 11–13	PRE-POST
Massey et al. (2025)	UK	Feasibility RCT of outdoor swimming for depression	Outdoor/nature-based swimming course	*N* = 87 participants	Youth aged 18 years	RCT
Duffy and Ashbullby (2025)	UK	Explore the impact of open-water swimming on student wellbeing	Wild swimming groups	*N* = 8	University students (age 20 to 24)	QUALI
Harper et al. (2025)	Norway and UK	Assess cold-water immersion course effects on adolescent mood	Outdoor cold-water immersion program	33 (in abstract)	Adolescents	PRE-POST
Mullooly and Colbert (2024)	Ireland/UK	Examine the relationships among cold-water immersion, resilience, self-efficacy, and mental toughness.	Community cold-water immersion groups	NR	Adolescent age	RCT
Silva et al. (2019)	Brazil	Evaluate swimming training effects on mental health and Cognition in children with ADHD	Sports center/training facility	36	Children (7–12 yrs)	RCT
Burlingham et al. (2022)	UK	Feasibility of sea swimming for depression and anxiety	Sea swimming groups	*N* = 53	Youth aged 18	PRE-POST

RCT, randomized controlled trial; QEXP, quasi-experimental study design; PRE-POST, pretest-posttest design without a control group; QUALI, Qualitative study; QUANTI, Quantitative study.

The reviewed studies were conducted across a diverse range of geographical contexts, predominantly in Europe (e.g., Burlingham et al., 2022; Mullooly and Colbert, 2024; Massey et al., 2025), Asia (Yu et al., 2024; Zulkifli and Bachtiar, 2025), and the Middle East (Köroglu and Yigiter, 2016; Hattabi et al., 2022). The United Kingdom and Ireland featured prominently, particularly in research exploring wild swimming, sea swimming, and open-water interventions (Huque and Yue, 2025; McDougall et al., 2022; Taylor et al., 2025; Massey et al., 2025; Burlingham et al., 2022; Duffy and Ashbullby, 2025). Nordic contexts were represented by studies conducted in Norway and the UK that examined cold-water immersion among adolescents (Harper et al., 2025). Asian countries contributed experimental evidence from China (Yu et al., 2024), Indonesia (Zulkifli and Bachtiar, 2025), and Turkey (Köroglu and Yigiter, 2016), while the Middle East and North Africa were represented by randomized controlled trials from Egypt and Tunisia focusing on adolescent health outcomes (Abdelrahman et al., 2024; Hattabi et al., 2022). Brazil contributed experimental research involving children with ADHD (Silva et al., 2019).

In line with the strict review’s inclusion criteria, the studies focused on adolescents and youth, although the exact age focus varied. Several studies explicitly targeted adolescents, including school-aged children and early adolescents aged approximately 11–18 years (Köroglu and Yigiter, 2016; Abdelrahman et al., 2024; Harper et al., 2025). Youth and young adults, including university students, pre-service teachers, and young people experiencing mental health challenges, were the focus of other studies (Zulkifli and Bachtiar, 2025; Taylor et al., 2025; Duffy and Ashbullby, 2025; Mullooly and Colbert, 2024). Although a small number of studies involved younger children (e.g., Yu et al., 2024; Silva et al., 2019; Mills et al., 2020), they were included because they contributed experimental evidence on mental health-related indicators relevant to developmental trajectories discussed in the review. Participants across the reviewed studies included school students, university students, pre-service teachers, young people experiencing depression or anxiety, and children with neurodevelopmental conditions. This diversity highlights the broad applicability of swimming interventions across populations with varying mental health needs. While some studies targeted clinically defined groups, others focused on general youth populations, reflecting both preventive and therapeutic applications of swimming for mental well-being.

Furthermore, the introductory finding of this review demonstrates that the methodological approaches varied considerably, reflecting the multidimensional nature of aquatic interventions. Twelve studies adopted experimental or quasi-experimental designs. Among these, randomized controlled trials compared aquatic exercise with alternative interventions or control conditions, such as aerobic exercise or usual care (e.g., Silva et al., 2019; Mills et al., 2020; Hattabi et al., 2022; Massey et al., 2025). In comparison, a single study employed a Quasi-Experimental Design to examine day-to-day fluctuations in mental health associated with cold-water swimming (Forsten and Wetherell, 2025). The rest of the intervention-based studies performed pre–post intervention to assess the effect of swimming-based intervention on adolescents and youth’s corresponding mental health indicators through comparing the baseline and post-test results (Köroglu and Yigiter, 2016; Burlingham et al., 2022; Yu et al., 2024; Harper et al., 2025; Zulkifli and Bachtiar, 2025).

Meanwhile, the remaining four studies employed qualitative research approaches to gain in-depth insight into participants’ lived experiences of swimming and engagement with blue spaces (Huque and Yue, 2025; McDougall et al., 2022; Taylor et al., 2025; Duffy and Ashbullby, 2025). Data were primarily collected through semi-structured, in-depth interviews, with some studies additionally drawing on observational or place-based contextual data. Participants included adolescents, young adults, university students, and adult recreational swimmers engaged in organized or informal wild, sea, or open-water swimming. Huque and Yue (2025) interviewed members of wild swimming groups to examine how collective swimming practices function as informal “ecologies of care,” focusing on relational support, shared vulnerability, and emotional well-being. McDougall et al. (2022) conducted interviews with freshwater wild swimmers to explore how place, environmental risk, and sensory engagement with water shaped perceptions of health and well-being. Taylor et al. (2025) interviewed adolescents and young people participating in sea swimming programs designed to support mental health, aiming to understand how swimming influenced emotional regulation, confidence, and engagement with care outside clinical settings. Duffy and Ashbullby (2025) focused on university students and used qualitative interviews to investigate how open-water swimming and cold-water immersion contributed to stress management, empowerment, and psychological resilience. Across these studies, qualitative findings consistently highlighted experiential and contextual mechanisms—such as social connection, connection to place, embodied challenge, and identity development, through which swimming and blue-space engagement were perceived to support mental health and well-being. These insights complement the experimental evidence by elucidating how and why aquatic interventions may be psychologically meaningful for adolescents and youth.

Regarding the types of interventions used in the 12 experimental studies synthesized in this review, the findings show that the nature of swimming interventions varied widely across studies, encompassing both structured and informal aquatic activities (see Table 6). Indoor pool-based swimming and hydrotherapy were commonly used in clinical or school-based experimental studies, particularly those involving children and adolescents with specific health or developmental conditions such as ADHD, autism spectrum disorder, or dysmenorrhea (Abdelrahman et al., 2024; Hattabi et al., 2022; Mills et al., 2020; Silva et al., 2019). These interventions were typically supervised, time-bound, and delivered within structured programs. In contrast, a substantial body of research focused on outdoor and open-water swimming, including sea swimming, cold-water immersion, freshwater wild swimming, and surf-related activities (Huque and Yue, 2025; McDougall et al., 2022; Forsten and Wetherell, 2025; Harper et al., 2025; Massey et al., 2025; Burlingham et al., 2022). These interventions emphasized interaction with natural blue environments and often incorporated social or community-based elements, such as group swimming or peer support. Participants in these studies were typically adolescents, youth, or young adults who voluntarily engaged in swimming activities, with mental health benefits assessed in real-world contexts rather than in controlled laboratory settings.

**Table 6:** Experimental studies, intervention, and key findings.

Study	Setting and intervention type	Intervention classification	Duration/frequency	Mental health indicator	Measuring tool	Key findings
Abdelrahman et al. (2024)	Aquatic exercise program *vs.* aerobic training	Academic/clinical intervention	aquatic exercise for 12 weeks	Quality of life	Menstrual distress questionnaire (MDQ), short form-36 (SF-36)	The aquatic exercise group showed significantly greater improvement in the physical and mental component scores of the SF-36 post-intervention compared with the aerobic and control groups (*p* < 0.001).
Zulkifli and Bachtiar (2025)	Swimming participation/training	Academic/educational intervention	14 weeks (i.e., one session of 2 h/week),	Cognitive function, mental Health	DASS-21 (depression, anxiety, stress scales)	Swimming intervention significantly reduced scores for depression, anxiety, and stress (all *p* < 0.05) compared to the control group.
Forsten and Wetherell (2025)	Regular cold-water sea swimming	Hybrid (self-directed nature exposure studied academically)	at least twice a week	Daily mental health indices, mood, stress	Ecological momentary assessment (EMA), WEMWBS (short form)	On swimming days, participants reported significantly higher positive affect and well-being and lower negative affect and perceived stress than on non-swimming days (*p* < 0.01).
Hattabi et al. (2022)	Swimming-based ADHD treatment	Academic/therapeutic exercise intervention	12 weeks (reported in the article)	ADHD core symptoms, behavioral problems	Conners’ parent rating scale-revised (CPRS-R), strengths and difficulties questionnaire (SDQ)	The swimming group showed significant reductions in inattention, hyperactivity/impulsivity, and total ADHD symptoms on the CPRS-R (*p* < 0.01) versus the control group.
Mills et al. (2020)	Hydrotherapy aquatic program	Academic/clinical intervention	8 weeks total (phased crossover)	Behaviors related to mental wellbeing (e.g., anxiety, irritability)	Repetitive behavior questionnaire (RBQ), child behavior checklist (CBCL)	Hydrotherapy led to a significant decrease in scores for irritability (*p* = 0.02) and stereotyped behaviors (*p* = 0.01) post-session compared to the control activity.
Yu et al. (2024)	Parent-accompanied structured swimming	Academic/developmental intervention	8 weeks, structured sessions	Intelligence (as a cognitive mental health indicator)	Wechsler preschool and primary scale of intelligence (WPPSI)	The parent-accompanied swimming group showed a significantly greater increase in Full-Scale IQ (*p* < 0.05) and Verbal IQ (*p* < 0.01) scores than the control group after 8 weeks.
Köroglu and Yigiter (2016)	Structured swimming program	Academic/educational intervention	2 h for 8 weeks.	Stress levels	Perceived stress scale (PSS)	Post-training stress scores were significantly lower in the swimming group compared to both pre-test scores and the control group’s post-test scores (*p* < 0.05).
Massey et al. (2025)	Outdoor group swimming course	Nature-based therapy (formalized)	8-session program	Depression severity, psychological wellbeing	Patient health questionnaire-9 (PHQ-9), warwick-edinburgh mental wellbeing scale (WEMWBS)	Feasibility study: outdoor swimming group showed a significant reduction in PHQ-9 scores (mean difference −5.7 points) and a significant improvement in WEMWBS scores (mean difference +6.2 points) at 12 weeks.
Harper et al. (2025)	Cold-water immersion course	Nature-based therapy (structured course)	half-day course	Mood states	Profile of mood states (POMS) questionnaire	Following the cold-water immersion course, adolescents reported a significant improvement in total mood disturbance (*p* < 0.001), with notable reductions in tension, depression, and anger.
Mullooly and Colbert (2024).	Cold-water immersion participation	Hybrid (recreational → therapeutic)	16 weeks	Mental health, resilience, self-efficacy	Mental health continuum-short form (MHC-SF), brief resilience scale (BRS), general self-efficacy scale (GSE)	Frequency of cold-water immersion was positively correlated with higher scores on mental wellbeing (*r* = 0.32, *p* < 0.01), resilience (*r* = 0.28, *p* < 0.05), and self-efficacy (*r* = 0.35, *p* < 0.01).
Silva et al. (2019)	Swimming training intervention	Academic/training intervention	12 weeks	Mental health parameters (e.g., anxiety), cognition	Child behavior checklist (CBCL), stroop color-word test	The swimming group demonstrated significant improvements in attention and cognitive flexibility (stroop test, *p* < 0.05) and reductions in internalizing problems on the CBCL (*p* < 0.05) compared with the control group.
Burlingham et al. (2022)	Sea-swimming program	Nature-based therapy (formalised)	8–12 sessions	Depression, anxiety, wellbeing	Hospital anxiety and depression scale (HADS), wemwbs	Post-intervention, 63% of participants met reliable improvement criteria for depression (HADS-D), and 71% for anxiety (HADS-A). wellbeing (WEMWBS) scores significantly increased (*p* < 0.001).

### 5.2 The association between water sport intervention and psychological well-being

Across the 12 experimental studies reviewed, a consistent pattern emerged demonstrating a positive association between swimming-based interventions and psychological and mental well-being among adolescents and youth. Controlled and quasi-experimental trials showed that structured swimming, aquatic exercise, hydrotherapy, and cold- or open-water swimming were associated with significant reductions in stress, anxiety, depressive symptoms, and behavioral difficulties, alongside improvements in mood, self-confidence, resilience, and overall quality of life. For instance, randomized controlled trials reported that aquatic exercise produced greater improvements in quality of life and psychological comfort than land-based aerobic exercise among adolescent females (Abdelrahman et al., 2024), while swimming training significantly reduced perceived stress levels among early adolescents (Köroglu and Yigiter, 2016). Experimental studies focusing on neurodevelopmental conditions further indicated that swimming and hydrotherapy interventions improved emotional regulation, attention, behavior, and psychosocial functioning in children and adolescents with ADHD and autism spectrum disorder (Hattabi et al., 2022; Mills et al., 2020; Silva et al., 2019).

Evidence from outdoor and cold-water swimming studies reinforced these findings, showing both short-term and sustained mental health benefits. Regular cold-water sea swimming was associated with daily improvements in mood and reductions in anxiety. It enhanced subjective well-being (Forsten and Wetherell, 2025), while feasibility trials of outdoor swimming as a nature-based intervention demonstrated clinically meaningful reductions in depressive symptoms and anxiety (Massey et al., 2025; Burlingham et al., 2022). Additional experimental work linked cold-water immersion and swimming participation with higher self-efficacy, resilience, and mental toughness, suggesting broader psychological gains beyond symptom reduction (Mullooly and Colbert, 2024; Harper et al., 2025). Collectively, these experimental findings indicate that swimming-based interventions exert beneficial effects across multiple dimensions of mental health, regardless of whether activities occur in indoor pools, therapeutic settings, or natural blue environments.

The four qualitative studies reviewed provide important interpretive depth, helping explain how and why these psychological benefits occur. Participants consistently described swimming, particularly wild, freshwater, and sea swimming, as producing immediate emotional relief, mood elevation, and a sense of calm, directly supporting the experimental evidence of reduced anxiety and stress (Huque and Yue, 2025; McDougall et al., 2022). Qualitative accounts emphasized swimming as a meaningful coping strategy for managing psychological distress, with young people reporting feelings of empowerment, accomplishment, and renewed self-belief after overcoming physical and environmental challenges such as cold water, waves, or perceived risk (Taylor et al., 2025; Duffy and Ashbullby, 2025). These experiences align closely with experimental findings related to increased resilience, self-confidence, and mental toughness.

Moreover, qualitative studies highlighted social and environmental mechanisms that are not easily captured in experimental designs. Group-based swimming fostered social connection, mutual support, and a sense of belonging, which participants identified as central to their improved emotional well-being (Huque and Yue, 2025). Engagement with natural blue spaces was also described as restorative, promoting emotional regulation, reflection, and a temporary escape from academic pressures and digital overload, thereby reinforcing mood improvements observed quantitatively (McDougall et al., 2022; Duffy and Ashbullby, 2025). Taken together, the qualitative evidence complements and strengthens the experimental findings by illustrating that emotional relief, mood elevation, empowerment, social connectedness, and connection to blue spaces are key pathways through which swimming-based interventions support psychological and mental well-being among adolescents and youth.

### 5.3 Age- and intervention-specific effects of swimming on psychological wellbeing

Synthesis of the 12 experimental studies indicates that the psychological and mental health effects of swimming-based interventions differ meaningfully across age groups, with adolescents and youth benefiting through partly distinct pathways and outcome profiles. Among adolescents, particularly those in early to mid-adolescence, swimming interventions were consistently associated with significant reductions in stress, anxiety, behavioral difficulties, and emotional dysregulation. School- and clinic-based trials demonstrated that structured swimming programs lowered perceived stress among students aged 11–13 and improved mood stability and emotional control during a sensitive developmental period characterized by heightened vulnerability (Köroglu and Yigiter, 2016). Similarly, randomized trials involving adolescents with neurodevelopmental conditions showed that swimming and hydrotherapy produced significant improvements in attention, emotional regulation, and psychosocial functioning compared with control or alternative conditions, indicating more potent effects on behavioral and emotional symptoms in younger populations (Hattabi et al., 2022; Mills et al., 2020; Silva et al., 2019). In adolescent females, aquatic exercise yielded greater improvements in quality of life and psychological comfort than land-based aerobic exercise, suggesting that water-based activity may be particularly effective for addressing stress-related and affective symptoms during puberty and early adolescence (Abdelrahman et al., 2024). Experimental studies focusing specifically on adolescents engaging in cold-water immersion further demonstrated short-term mood elevation and reductions in negative affect. However, these effects were often described as immediate and state-based rather than sustained trait-level changes (Harper et al., 2025).

In contrast, studies focusing on youth and young adults, such as university students, pre-service teachers, and older adolescents transitioning into adulthood, showed that swimming interventions exerted more potent effects on broader psychological well-being, self-efficacy, resilience, and depressive symptoms than on behavioral regulation. Quantitative studies among young adults found that regular swimming was associated with improvements in cognitive functioning, reduced psychological distress, and enhanced mental clarity, reflecting outcomes aligned with academic and occupational demands characteristic of this life stage (Zulkifli and Bachtiar, 2025). Outdoor and cold-water swimming interventions in youth populations demonstrated statistically significant reductions in depression and anxiety, alongside increases in self-confidence and perceived vitality, particularly when sustained over several weeks (Forsten and Wetherell, 2025; Massey et al., 2025; Burlingham et al., 2022). These findings suggest that while adolescents may benefit most through symptom reduction and emotional regulation, youth appear to experience more substantial gains in positive mental health indicators, including resilience, autonomy, and psychological flourishing.

Further, the literature review found that the differences were also clear when comparing the types of swimming interventions, not only in mental health outcomes but also in the environmental contexts and pressures associated with each setting. Across various age groups, indoor pool-based and therapeutic swimming interventions showed strong effects on clinical and subclinical symptoms, including stress, anxiety, ADHD-related behaviors, and emotional dysregulation, particularly in children and adolescents (Hattabi et al., 2022; Mills et al., 2020; Silva et al., 2019). These interventions occurred in highly controlled aquatic environments, mainly municipal or clinical pools, where water quality, temperature, and exposure risks were managed. Consequently, environmental impacts were minimal and mostly related to resource-intensive infrastructure demands, such as energy use for heating, chemical treatment, and water circulation. None of these studies specifically evaluated environmental sustainability outcomes. However, their use of built aquatic facilities suggests relatively low direct ecological disturbance and higher indirect environmental costs due to long-term resource use.

In contrast, outdoor, open-water, and cold-water swimming interventions, conducted in seas, lakes, rivers, and coastal areas, proved particularly effective in improving mood, reducing depression, building self-efficacy, and enhancing resilience, especially among youth and older adolescents. Randomized controlled trials and longitudinal studies showed that nature-based swimming interventions led to larger improvements in depressive symptoms and sustained wellbeing than traditional indoor programs. This highlights the additional psychological benefits of immersion in natural water environments (Massey et al., 2025; Forsten and Wetherell, 2025). However, these benefits emerged in ecologically dynamic and vulnerable settings, which several studies acknowledged as areas of potential environmental pressure. For example, feasibility trials and exploratory interventions in coastal and freshwater locations identified challenges such as seasonal overcrowding, changes in water quality, safety risks, and ecological sensitivity, especially in popular wild swimming spots (Massey et al., 2025; McDougall et al., 2022; Burlingham et al., 2022).

Cold-water immersion activities were specifically linked to increased mental toughness and self-efficacy, outcomes less commonly reported in indoor swimming studies (Mullooly and Colbert, 2024). These interventions often took place in minimally altered natural waters, enhancing participants’ feelings of authenticity and connection to nature. At the same time, studies highlighted the need for careful environmental stewardship, as repeated group immersion in delicate aquatic ecosystems could lead to shoreline erosion, habitat disturbance, and increased waste or pollution if not managed properly (McDougall et al., 2022). While none of the reviewed studies systematically measured ecological impact, several acknowledged the tension between expanding access to blue-health interventions and preserving the long-term sustainability of aquatic environments.

The four qualitative studies supported and contextualized these quantitative age- and intervention-related differences while providing clearer insights into the relationship between environment and health. Adolescents described swimming, particularly in the sea or in groups, as a source of immediate emotional relief, mood stabilization, and a sense of safety during periods of distress (Taylor et al., 2025). Participants in wild and sea swimming studies often stressed the value of respecting the environment, being aware of tides, weather, and water conditions, and engaging in informal practices of care, such as minimizing disturbance and following local norms (Huque and Yue, 2025). These narratives present blue spaces not just as therapeutic settings but as active ecosystems, where mental health benefits are connected to ethical relationships with the environment.

Youth participants focused on empowerment, identity reconstruction, and boosted self-belief, echoing quantitative findings of increased resilience and self-efficacy in older groups (Duffy and Ashbullby, 2025). Across qualitative studies, involvement in natural blue spaces was frequently linked to improved mood, emotional release, and a sense of renewal. This provides insight into why outdoor and cold-water swimming interventions had stronger effects on depression and well-being than indoor programs (Huque and Yue, 2025; McDougall et al., 2022). Simultaneously, participants and authors raised concerns about overuse, commercialization, and unequal access, particularly as growing interest in wild swimming could put more stress on already burdened aquatic environments.

Participants also mentioned social connection and shared challenges as vital elements, suggesting that group-based and nature-oriented swimming could be especially beneficial for youth seeking autonomy, belonging, and meaning during their transition to adulthood. However, these social aspects were also seen as potential sources of environmental strain when group sizes increased without appropriate oversight, infrastructure, or conservation planning (McDougall et al., 2022).

Unfortunately, the present review, which combines experimental and qualitative evidence, shows that both age and the type of intervention environment influence the mental health benefits of swimming, while also revealing clear differences in environmental exposure and ecological pressure. Indoor and therapeutic programs provide emotionally protective and clinically effective interventions with limited direct ecological impact but higher infrastructure needs. In contrast, outdoor, cold-water, and nature-based swimming have a stronger positive effect on youth wellbeing, resilience, and mental health, yet depend on ecologically sensitive blue spaces that may face growing pressures from increased human activity. This mix shows a significant gap in the existing research: despite strong evidence for mental health benefits, systematic assessment of environmental impact and sustainability is mostly missing. Future research should include ecological monitoring, place-based ethics, and sustainable access, alongside mental health outcomes.

## 6 Discussion

The findings of this systematic review provide substantial evidence that swimming interventions significantly improve psychological and mental well-being among adolescents and youth. Experimental studies consistently demonstrated reductions in stress, anxiety, and depressive symptoms, as well as enhancements in mood, resilience, and self-efficacy across participants aged 12 to 25 (Abdelrahman et al., 2024; Silva et al., 2019; Köroglu and Yigiter, 2016; Zulkifli and Bachtiar, 2025). Notably, the magnitude and nature of these effects varied by age group. Adolescents exhibited greater reductions in stress and behavioral symptoms, particularly when engaging in structured aquatic interventions such as swimming-based therapies for ADHD or hydrotherapy for children with autism spectrum disorder (Hattabi et al., 2022; Mills et al., 2020; Silva et al., 2019). In contrast, young adults experienced more pronounced improvements in mood, emotional regulation, and overall psychological wellbeing, especially in the context of open-water or group-based swimming interventions (Forsten and Wetherell, 2025; Massey et al., 2025; Duffy and Ashbullby, 2025). These age-specific differences suggest that developmental stage influences responsiveness to swimming interventions, likely reflecting the interplay between social, cognitive, and emotional maturation.

The type and setting of swimming interventions further moderated outcomes. Outdoor, cold-water, and open-water swimming demonstrated the most decisive influence on psychological health, possibly due to the synergistic effects of natural environmental exposure, multisensory stimulation, and social interaction (Huque and Yue, 2025; McDougall et al., 2022; Christie and Elliott, 2025). By contrast, indoor pool-based or parent-accompanied swimming programs were associated with moderate improvements, particularly in cognitive, motor, and physiological outcomes among younger adolescents (Abdelrahman et al., 2024; Yu et al., 2024; Tavakolizadeh et al., 2012). This differentiation aligns with prior research emphasizing the unique psychological benefits of “blue space” engagement, whereby immersion in natural aquatic environments fosters emotional relief, mood elevation, and a sense of mastery that is less accessible in controlled indoor settings (Dawe et al., 2025; Murray and Fox, 2021; Oliver et al., 2023). Consequently, the findings underscore that intervention context and design are critical determinants of effectiveness and should be carefully considered when implementing swimming-based programs for mental health promotion.

Qualitative studies within the review provided valuable insights into the mechanisms underlying these effects. Participants consistently reported feelings of emotional relief, invigoration, and increased self-confidence, often linked to group dynamics, peer support, and interactions with natural environments (Taylor et al., 2025; Duffy and Ashbullby, 2025; Huque and Yue, 2025; McDougall et al., 2022). These subjective experiences complement quantitative findings by highlighting how psychological benefits are mediated not only by physical exertion but also by social cohesion, sensory engagement, and perceived mastery of challenging aquatic tasks (Christie and Elliott, 2025; Dawe et al., 2025). Such integrated evidence strengthens the argument that swimming interventions operate through multifaceted pathways, combining physiological, cognitive, and psychosocial mechanisms to enhance mental wellbeing.

By synthesizing experimental and qualitative evidence, this review refines the existing literature on aquatic interventions for adolescents and youth. Prior reviews often focused on clinical populations, older adults, or broad age ranges, limiting their applicability to general youth populations (Sumartana and Setiaji, 2025; Wang et al., 2025; de Oliveira et al., 2019). In contrast, the current review demonstrates that both the developmental stage and type of swimming intervention are critical determinants of psychological outcomes, offering nuanced guidance for designing targeted mental health programs. Outdoor and cold-water swimming emerge as particularly potent interventions for young adults. In contrast, structured indoor swimming remains highly effective for younger adolescents, highlighting the importance of aligning intervention characteristics with developmental needs. Overall, these findings extend the evidence base for swimming as a mental health intervention, providing actionable insights for educators, practitioners, and policymakers seeking to mitigate the rising mental health challenges among adolescents and youth (Ferreira et al., 2024; Szabo et al., 2019; Almási et al., 2021).

## 7 Conclusion

This systematic review provides compelling evidence that swimming interventions exert a significant and positive impact on the psychological and mental wellbeing of adolescents and youth. The synthesized findings demonstrate that both age and the type of swimming intervention critically influence outcomes, with structured indoor swimming particularly effective for younger adolescents, and outdoor, open-water, or cold-water swimming eliciting pronounced benefits for young adults. Qualitative insights further underscore that these interventions enhance mental health not only through physical activity but also via emotional relief, mood elevation, social connectedness, and engagement with natural environments. By integrating experimental and qualitative evidence, this review clarifies the mechanisms through which swimming promotes emotional regulation, resilience, and overall psychological functioning, addressing a critical gap in youth-focused research. These findings provide strong empirical justification for incorporating swimming-based programs into mental health promotion strategies for adolescents and young adults, highlighting the potential of targeted aquatic interventions to mitigate the rising burden of youth mental health challenges globally.

### 7.1 Policy and practice implications

The findings of this systematic review underscore the significant potential of swimming and aquatic-based interventions to enhance mental health and psychological wellbeing among adolescents and youth. For practitioners, including physical education teachers, school counselors, and youth mental health professionals, these results highlight the value of integrating structured aquatic activities into school curricula, after-school programs, and community-based interventions. Programs that incorporate pool swimming, open-water immersion, or guided hydrotherapy can serve as practical tools for reducing stress, anxiety, and depressive symptoms, while also promoting resilience, self-efficacy, and emotional regulation among young people (Huque and Yue, 2025; Massey et al., 2025; Abdelrahman et al., 2024).

For policymakers, the evidence supports prioritizing funding and infrastructure to expand access to aquatic facilities, particularly in urban and underserved communities where sedentary lifestyles and mental health risks are elevated. Policies that incentivize youth participation in water-based physical activity, such as subsidized swimming programs, community swimming events, or partnerships with recreation centers, could have substantial public health benefits. Moreover, public health campaigns and school-based initiatives should emphasize the dual benefits of aquatic activity for both physical and mental health, framing swimming as a preventive strategy for mental health challenges in adolescents and young adults (Duffy and Ashbullby, 2025; Mullooly and Colbert, 2024).

For researchers and intervention designers, the differential effects observed across age groups and intervention types suggest that programs should be tailored developmentally and contextually, with outdoor, open-water, or nature-based interventions appearing particularly promising for mood elevation and stress reduction (Forsten and Wetherell, 2025; Burlingham et al., 2022). Future policy and practice should encourage the adoption of evidence-informed aquatic programs that consider duration, intensity, social engagement, and environmental context to maximize psychological benefits. Collectively, these findings advocate for a strategic, multisectoral approach that leverages swimming and aquatic interventions to mitigate the growing mental health burden among youth populations.

### 7.2 Limitations and future research directions

Despite the strengths of this systematic review, several limitations should be acknowledged. First, the restriction to studies published in English may have excluded relevant research conducted in other languages, potentially introducing publication bias and limiting the generalizability of the findings globally. Future reviews should consider multilingual searches to capture a more comprehensive international perspective. Second, the focus on adolescents and youth aged 12–25, while essential for demographic specificity, means that insights from younger children or older adults, who may also benefit from aquatic interventions, were omitted; future studies could explore developmental differences across broader age ranges better to understand the lifespan effects of swimming on mental health. Third, while the inclusion of diverse study designs enhanced the quality of the review, it also introduced heterogeneity in intervention types, outcome measures, and study quality, which constrained the ability to conduct meta-analytic synthesis. Future research should aim for standardized outcome measures, intervention protocols, and robust experimental designs, such as large-scale randomized controlled trials, to improve comparability of evidence. Fourth, interventions in the included studies varied widely in type, duration, intensity, and setting (e.g., pool versus open water), limiting the ability to identify optimal intervention characteristics. Subsequent research should systematically compare different aquatic modalities and contexts to delineate which types of swimming interventions are most effective for specific mental health outcomes. Fifth, the review included only studies with a minimum duration of 2 weeks, which may have excluded potentially informative acute or single-session studies that capture immediate psychological effects; future research could examine both short-term and long-term effects to provide a more nuanced understanding of intervention timing and dose–response relationships. Lastly, although qualitative studies provided valuable insights into participants’ lived experiences and subjective benefits, their inclusion was limited, suggesting the need for more in-depth qualitative exploration to understand mechanisms such as emotional relief, social connectedness, and the role of natural aquatic environments in supporting mental wellbeing. By addressing these limitations, future research can build a more robust, globally relevant, and developmentally sensitive evidence base to inform the design and implementation of swimming-based mental health interventions for young populations.

## Data Availability

The original contributions presented in the study are included in the article/Supplementary material, further inquiries can be directed to the corresponding author.
