# Supplementary material for: Blue health among children, adolescents, and youth psychological well-being: a systematic review of swimming and aquatic therapy for mental health
Source: Front Psychol. 2026 Jan 13;16:1732568. doi: 10.3389/fpsyg.2025.1732568 (PMC12834781; doi:10.3389/fpsyg.2025.1732568)
Supplement: Supplementary file 1 [file Table_1.docx]

**ADDITIONAL FILE**

# **Supplementary Table A. Swimming and Aquatic-based interventions vs control on the main outcome of the included studies**

The GRADE approach was applied to the pooled analysis of 12 trials investigating the effects of swimming intervention compared to a control intervention on the main outcome of the study. Pooled standardised effect size 0.15, 95% CI 0.05 to 0.25.

| **Overall result:** Swimming activity interventions probably improve mental health among children, adolescents and youths. | |
| --- | --- |
| **Level of evidence:** Moderate certainty  We are moderately confident in the effect estimate. The true effect is likely to be close to the estimate of the effect, but there is a possibility that it is substantially different. Further research is likely to have an important impact on our confidence in the estimate of effect and may change the estimate. | |
| **Study limitations** | We downgraded the evidence by one level as 10/12 (83%) of studies in the meta-analysis had a PEDro score <6/10. |
| **Imprecision** | We did not downgrade the evidence due to imprecision as the meta-analysis had 1,560 participants analysed. |
| **Inconsistency of results** | We did not downgrade the evidence due to heterogeneity of included studies as the heterogeneity between trials was small. Additionally, most of the comparisons in the meta-analysis were in the same direction (11/12, 91.7%). |
| **Indirectness of evidence** | Since we only included similar studies in terms of population, intervention, comparator and outcome, we did not downgrade the evidence based on this criterion. |
| **Publication bias** | We did not downgrade the evidence for publication bias. Additional sensitivity analysis where small studies (n<50) were excluded from the meta-analysis showed that the effect estimate is reasonably stable in the absence of small studies (pooled standardised effect size 0.12, 95% CI: 0.01 to 0.23). |

Shading indicates the items that were downgraded.

**Supplementary Table B.** Summary of findings (GRADE).

| **Certainty assessment** | | | | | | |
| --- | --- | --- | --- | --- | --- | --- |
| **No. of participants (studies)** | **Risk of bias** | **Inconsistency** | **Indirectness** | **Imprecision** | **Other** | **Overall certainty of evidence** |
|  |  |  |  |  |  |  |
| 1,560 (12 studies) | serious^a^ | not serious^b^ | serious^c^ | not serious | none | ⨁⨁◯◯ LOW |

Explanations:

a. Most studies did use appropriate instruments to measure mental health prevalence and did report how participants were recruited. Some studies had issues related to sample size and sample coverage.

b. Even though *I*^2^=90%, studies yielded similar results, with the point estimates of most studies ranging from 5 to 25% and showing an overlap of CIs. High I^2^ values are expected in a meta-analysis of prevalence studies, with limited significance in this context. In addition, most probable cause of statistical heterogeneity is the difference in study population, factor that has been penalized in the assessment of indirectness domain.
